# Keyword parsimony—lessons from a scoping review

**DOI:** 10.1186/s13643-021-01784-5

**Published:** 2021-08-13

**Authors:** Helen Buchanan, Karen Grimmer

**Affiliations:** 1 Division of Occupational Therapy, Department of Health and Rehabilitation Sciences, Faculty of Health Sciences, University of Cape Town, Cape Town, South Africa; 2 Division of Physiotherapy, Department of Health and Rehabilitation Science, Faculty of Medicine and Health Science, Stellenbosch University, Cape Town, South Africa

We recently completed a systematic scoping review on return to work after hand injury which has been accepted for publication in the *Journal of Hand Therapy* [1]. We searched four large databases (PubMed, EbscoHost, Scopus, and Scielo), by applying appropriate Boolean operators and a broad list of search terms [2, 3]. Our review aimed to comprehensively identify the current body of research evidence for factors associated with successful work-related transitions following any type of hand injury. From 259 potentially relevant articles, we included 38 primary studies. We excluded systematic reviews, as our intention was to establish a comprehensive body of primary evidence. We cross-checked the primary studies in the excluded systematic reviews against our included primary studies and included relevant primary studies that we had not already identified in our search. We also hand searched the reference lists of the included primary studies for other relevant articles.

Our experience of identifying relevant articles for the scoping review proved to be time-consuming and frustrating. We identified almost as many appropriate articles through handsearching, as we did from the database searches themselves. For instance, 10 relevant primary articles were identified from the reference lists of the excluded systematic reviews, that we had not already found in our primary searches. Given these challenges, we cannot say with certainty that we located all relevant articles in our area of interest.

After completing the review, we attempted to understand why we had had this experience. As the construction of a comprehensive search strategy is integral to the efficient completion of a comprehensive systematic review [2, 3], the only explanation we could provide was that the keywords we used in our search strategy must have been deficient. We thus collated the keywords from the included papers and determined the frequency with which they had been reported. We identified not only a lesson for unwary reviewers in the area of return to work after hand injury, but also an opportunity for researchers in this area to improve the way their research might contribute to the body of scientific evidence.

There was little commonality in the keywords, even when papers described the same condition. From the 38 included papers, we identified 135 unique keywords with the most frequent being “work” (used 38 times in 24 papers), “hand” (used 23 times in 17 papers), “return” (used 16 times in 15 papers), and “disability” (used 12 times in 11 papers). We described these keywords diagrammatically (Fig. 1) to show the range and frequency of words used, their variability, and potential relevance to local contexts only (note the use of a country-specific acronym, WSIB (Workplace Safety and Insurance Board)). Five articles provided no keywords at all.

**Fig. 1:**
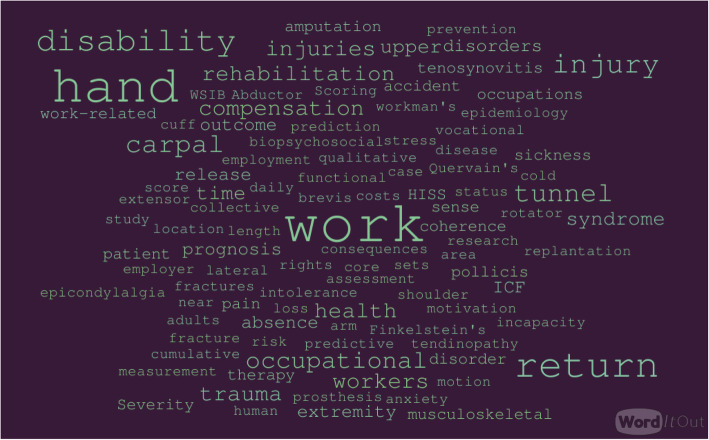
Word cloud of keywords

Our findings highlight opportunities for researchers in hand rehabilitation to collaborate and develop an agreed list of common keywords, that will ensure inclusion of their research in future systematic reviews. Hand rehabilitation, and return to employment, is integral to health [4, 5] and quality of life [6, 7]. A comprehensive defensible body of evidence is essential to ensure that people with hand injuries are rehabilitated in the best possible manner.

## Data Availability

Data are available in our scoping review (reference [1]).

## References

[1] Buchanan H, van Niekerk L, Grimmer K. Work transitions after hand injury: a scoping review. J Hand Ther. 2020:S0894-1130(20)30186-1. 10.1016/j.jht.2020.10.007 Online ahead of print. 10.1016/j.jht.2020.10.007 33250399

[2] Khalil H, Peters M, Godfrey C (2016). An Evidence-Based Approach to Scoping Reviews. Worldviews Evid-Based Nurs.

[3] Moher D, Liberati A, Tetzlaff J, Altman DG, The PRISMA Group (2009). Preferred Reporting Items for Systematic Reviews and Meta-Analyses: The PRISMA Statement. PLoS Med.

[4] Hergenrather KC, Zeglin RJ, McGuire-Kuletz M, Rhodes SD (2015). Employment as a social determinant of health: a systematic review of longitudinal studies exploring the relationship between employment status and physical health. Rehabil Res Pol Educ.

[5] Rueda S, Chambers L, Wilson M, Mustard C, Rouke SB, Bayoumi A, Raboud J, Lavis J (2012). Association of returning to work with better health in working-aged adults: a systematic review. Am J Public Health.

[6] Cederlund RI, Ramel E, Rosberg HE, Dahlin LB (2010). Outcome and clinical changes in patients 3, 6, 12 months after a severe or major hand injury - can sense of coherence be an indicator for rehabilitation focus?. BMC Musculoskelet Disord.

[7] Ramel E, Rosberg HE, Dahlin LB, Cederlund RI (2013). Return to work after a serious hand injury. Work.

